# Diagnosis of Breast Masses from Dynamic Contrast-Enhanced and Diffusion-Weighted MR: A Machine Learning Approach

**DOI:** 10.1371/journal.pone.0087387

**Published:** 2014-01-31

**Authors:** Hongmin Cai, Yanxia Peng, Caiwen Ou, Minsheng Chen, Li Li

**Affiliations:** 1 School of Computer Science and Engineering, South China University of Technology, Guangzhou, People’s Republic of China; 2 State Key Laboratory of Oncology in South China, Collaborative Innovation Center of Cancer Medicine, Sun Yat-sen University Cancer Center, Guangzhou, People’s Republic of China; 3 Department of Radiology, The Third Affiliated Hospital of Sun Yat-sen University, Guangzhou, People’s Republic of China; 4 Key Laboratory of Construction and Detection of Guangdong Province, Southern Medical University, Guangzhou, People’s Republic of China; Chinese Academy of Sciences, China

## Abstract

**Purpose:**

Dynamic contrast-enhanced magnetic resonance imaging (DCE-MRI) is increasingly used for breast cancer diagnosis as supplementary to conventional imaging techniques. Combining of diffusion-weighted imaging (DWI) of morphology and kinetic features from DCE-MRI to improve the discrimination power of malignant from benign breast masses is rarely reported.

**Materials and Methods:**

The study comprised of 234 female patients with 85 benign and 149 malignant lesions. Four distinct groups of features, coupling with pathological tests, were estimated to comprehensively characterize the pictorial properties of each lesion, which was obtained by a semi-automated segmentation method. Classical machine learning scheme including feature subset selection and various classification schemes were employed to build prognostic model, which served as a foundation for evaluating the combined effects of the multi-sided features for predicting of the types of lesions. Various measurements including cross validation and receiver operating characteristics were used to quantify the diagnostic performances of each feature as well as their combination.

**Results:**

Seven features were all found to be statistically different between the malignant and the benign groups and their combination has achieved the highest classification accuracy. The seven features include one pathological variable of age, one morphological variable of slope, three texture features of entropy, inverse difference and information correlation, one kinetic feature of SER and one DWI feature of apparent diffusion coefficient (ADC). Together with the selected diagnostic features, various classical classification schemes were used to test their discrimination power through cross validation scheme. The averaged measurements of sensitivity, specificity, AUC and accuracy are 0.85, 0.89, 90.9% and 0.93, respectively.

**Conclusion:**

Multi-sided variables which characterize the morphological, kinetic, pathological properties and DWI measurement of ADC can dramatically improve the discriminatory power of breast lesions.

## Introduction

The development of noninvasive methods of tissue characterization that could be applied early in the course of diagnosis to assess risk and to guild subsequent treatment would allow clinicians to tailor therapy on an individual. Conventional magnetic resonance imaging (MRI) of the breast has proven to be less successful than expected [1]. Breast MRI has demonstrated a high sensitivity, but with the shortcoming of varying specificity, reported to be from 37% to 97% [2], [3], [4], and therefore multiple biopsies tests have to be conducted as supplementary. Recently, more specialized methods, including dynamic contrast-enhanced magnetic resonance imaging (DCE-MRI) and diffusion-weighted magnetic resonance imaging (DW-MRI), have advanced to the point where they provide quantitative measurements of tissue properties that are highly related to the assessing of tumor progression and/or responses [1], [2], [5], [6], [7]. DW MRI was designed to reflect water movement within tissues by measuring the degree of random molecular motion and quantify such movement with apparent diffusion coefficient (ADC) value. Recent studies [8], [9], [10], [11], [12], [13] found that the ADC is significantly lower in malignant tumors than in benign breast lesions or normal tissue in DW MRI. This special observation is mainly due to a high cell density, caused by an increased restriction of the extracellular matrix and an increased fraction of the signal from intracellular water [8], [11], [14].

The advances in imaging techniques allow for the possibility to investigate the diagnostic performance by combining the merits of different image modalities. Such investigation is promising in clinical diagnosis by reducing inter-observer biases in interpretation of the images [15], [16], and by shortening the diagnosis time [17]. For example, it has been shown that the morphological features in breast MRI as adjunct diagnostic criteria can improve the specificity without significantly reducing the sensitivity [18], [19], [20]. Combining morphological characteristics with enhancement kinetics can also improve the diagnostic performance of breast lesion interpretation [21]. Yabuuchi et al. [22] reported a high accuracy in enhancing breast masses through the combination of DWI and DCE-MRI features.

However, there are few researches on investigation of the combinational performance of both MRI and DWI in discriminating of pathologically verified breast masses. In the current study, we retrospectively investigated the potential discriminatory power of image features estimated from both of DWI and DCE-MRI. Four distinct groups of features were estimated to comprehensively characterize the image in a multi-sided way. To remove the redundancy as well as to increase the diagnosis capabilities of the features, a hybrid feature selection scheme was conducted on the four feature groups and a pathological variable group. The resulted seven features, including one for pathology, one for morphology, three for texture and one for kinetic characteristics, were widely tested by standard classification models to demonstrate their combinational prognostic capabilities.

## Materials and Methods

### Patients and Lesions

The study comprised of 234 female patients from - Sun Yat-sen University Cancer Center (Guangzhou, China P. R.). The consecutive patients (mean age, 46.2 years ±10.9 [standard deviation]; range, 18–78 years) were enrolled into the study between September 2008 and December 2011. This study was approved by the Ethics Committee of Sun Yat-sen University Cancer Center, and all patients signed consent to participate in this study.

There were 85 benign lesions and 149 malignant lesions. Enrollment of the lesions abided by a strict inclusion criteria: (a) MR imaging was performed on a 1.5 T superconductive magnetic system (GE, Signa, HDx), with a bilateral, dedicated four-channel phased-array breast coil in its prone position.; (b) both DCE-MR imaging and DW MR imaging sequences were performed; (c) diagnosis was confirmed following a pathological analysis after core-needle biopsy or surgical excision (248 lesions), or lesion stability was confirmed at a minimum follow-up of 2 years (27 lesions); (d) lesions were presented as a mass according to the BI-RADS MRI lexicon; and (e) patients had not had a biopsy or received therapy before MR examination. Table 1 shows the distribution of histopathological findings of all analyzed lesions.

**Table 1:** Histopathology of benign and malignant breast lesions.

Tumor group	Number	Percentage
**Malignant lesions**	**149**	**63.68**
Invasive ductal carcinoma	120	51.3
Intraductal carcinoma	17	7.26
Ductal carcinoma in situ	4	1.7
Mucinous carcinoma	3	1.28
Medullary carcinoma	1	0.43
Others	4	1.71
**Benign lesions**	**85**	**36.32**
Fibroadenoma	26	11.11
Fibrocystic changes	24	10.26
Fibroadenosis	3	1.28
Intraductal papilloma	4	1.7
Hyperplasia	3	1.28
Phyllodes tumor	2	0.85`
Adenomyosis epithelioma	1	0.43
Inflammation	1	0.43
Follow-up	21	8.97

### Features Estimated from MR Images

To fully characterize the pictorial properties of the lesions, four different groups of features were estimated from the image to portray the distinct and remarkable features related to lesions, and another one group included the patients’ pathological test results. The five groups produced twenty-eight measurements (called *feature* herein) for each lesion. All the features obtained were extracted by two radiologists with ten years’ experience in interpreting breast MR. They were blind to the histological results on current patients. The images were assessed independently and all disagreements were resolved through consensus. All images were analyzed on a workstation (Centricity Radiology RA 600 V 7.0, GE, USA). The four groups of features were summarized below:

1. **Kinetic features:** The shape of time-signal intensity curve has been shown to be an important criteria in differentiating benign from malignant breast lesions [23]. Both of the early-phase enhancement and the signal enhancement ratio (SER) [24] were estimated to represent the kinetic behavior of the lesion signal intensity of lesion before and after the injection of Gd-DTPA. They are defined as:

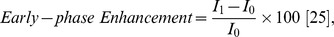


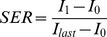
where 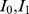, and 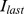 represent the signal intensity in the pre-contrast, the first post-contrast and the last images, respectively. The morphology and enhancement kinetic features were also investigated to determine their diagnostic performance to differentiate between malignant and benign lesions that present as mass versus non-mass types [26], [27].

**Figure 1:**
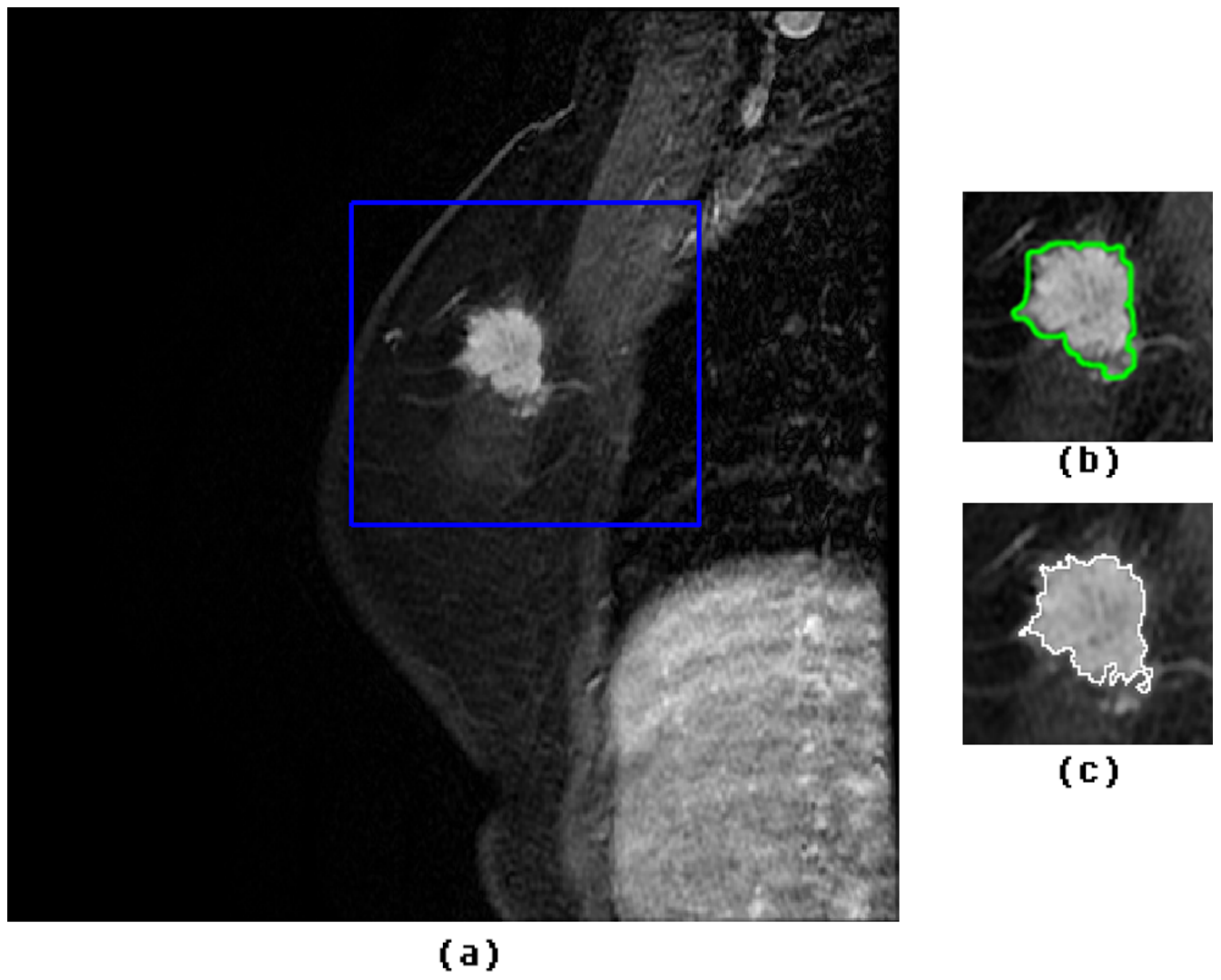
Segmentation of a sample breast lesion on MRI, confirmed as Invasive ductal carcinoma, for a 50 year old woman. (a) Area including a suspicious breast lesion is highlighted by a blue rectangle; (b) Initial segmentation result on (a) by using FCM-based method; (c) Final segmented lesion after GVF snake model initialized from (b).

**Figure 2:**
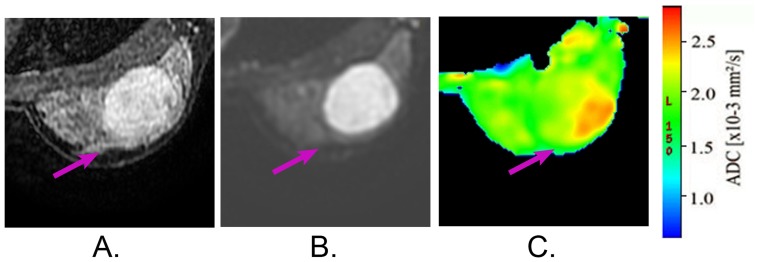
A sample image of fibroadenoma for a 28 year woman. (a) Raw dynamic contrast-enhanced MR image on lesion, which exhibits high signal intensity. The mass-like enhancement area is marked by purple arrow and the lesion; (b) Raw Diffusion-weighted MR image (b = 800 s/mm^2^); (c) Calculated ADC map from (b). Lesion area exhibits with light green (pointed in purple arrow), implying a high ADC value. ADC measured in this lesion is 1.91×10^−3^ s/mm^2^.

1. **Morphological Features:** The manually identified lesion was further segmented to have its contours. The segmentation used a two-step approach to incorporate fuzzy c-means (FCM) clustering [28] and gradient vector flow (GVF) snake algorithm [29]. Once the lesion was segmented, eleven morphological features were calculated to quantify its morphological characteristics. The lexicon by using morphology characteristics, such as shape and margin categories, have long been adopted in discrimination of breast lesion [30]. Its diagnostic capability were also widely studied by combining it with various factors, such as kinetic descriptor [31], texture features [26], [32] and DWI [33]. In the current study, eleven morphological features include compactness, spiculation, extent, elongation, solidity, circularity, entropy of radial length distribution, fractal, heterogeneity, area, and eccentricity were borrowed to serve as morphological characterization. Inclusion of the eleven features followed a strict criteria: either they are used in clinical practice or have been reported to be effective [30], [34]. An illustrate examples is shown in Figure 1.
2. **Texture Features:** The textural attributes evaluated via GLCM method were combined with morphologic descriptors in DCE-MRI to achieve a nice discrimination power [30], [31], [32], [35]. It has also been reported that MRI texture features are significantly associated with breast tumor subtype and neoadjuvant therapy response. Thirteen texture features were estimated on the segmented lesion through its gray level co-occurrence matrix (GLCM) [36]. The texture features included: angular second moment, contrast, correlation, inverse difference moment, sum average, sum variance, sum entropy, entropy, difference average, difference variance, difference entropy, information measure of correlation 1, and information measure of correlation 2 [36]. This feature group is widely used in field of pattern recognition, such as handwriting discrimination [37], [38], and medical image analysis [39], [40]. Readers can refer to File S1 for the rigorous mathematical definitions of the pictorial features.
3. **DWI Features:** The apparent diffusion coefficient (ADC) value was used to quantify the Diffusion weighted (DW) MRI. Firstly, the region of interests (ROIs) were manually drawn on the diffusion-weighted images (b = 800 s/mm2) (Figure 2) by carefully inspecting the regions with high signal. ROIs that were larger than 20 mm^2^ were considered meaningful and therefore retained for further analysis [41]. Then the DWI intensity for each lesion was dichotomized into low and high values by that of the corresponding background breast tissue. Finally, the mean ADC values were then obtained to serves as the quantification of the DWI characteristics.

The status of breast masses enrolled in the study was all verified in histopathology, or confirmed by at least two years of follow-up subsequently. Therefore, the features aforementioned, coupling with the lesion status, can be considered as a binary classification problem.

### Diagnostic Feature Selection

Univariate analysis is limited since it ignores the role of combinational potentials which could provide a good classification. Therefore, we conducted firstly on the selection of a subgroup of informative variables that were able to distinguish malignant lesions from benign ones. This process is known as feature subset selection (FSS) [42], [43], [44], [45].

**Figure 3:**
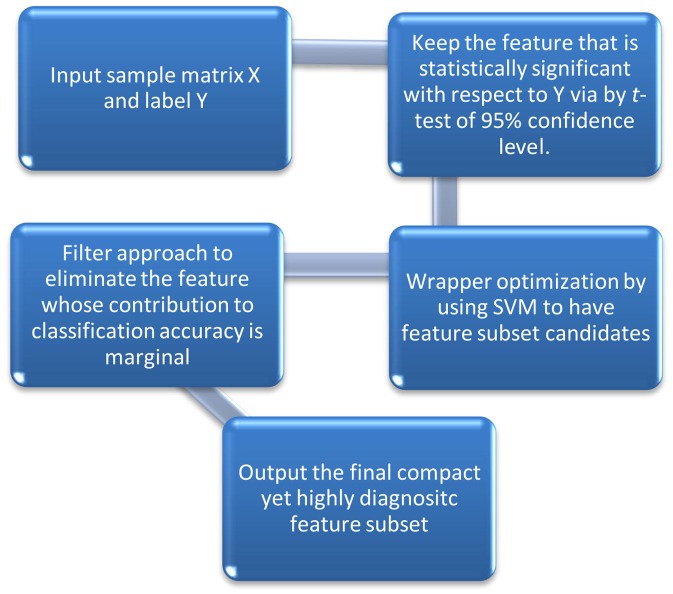
The workflow of the hybrid FSS scheme to have a compact but informative feature subset.

Feature selection algorithms usually fall into two categories [42]: filter and wrapper methods. Filter selects subsets of features as a preprocessing step, independently of the chosen predictor. In comparison, wrapper uses a base classifier to score subsets of features according to their predictive power. In many cases, wrapping with classical classifiers such as Support Vector Machine [44], Naive Bayes [46] and Nearest Neighbors produce comparable performance [47]. The wrapper has the advantage of better performance; however, its usage in biomedical area is limited due to its high computational cost [42]. To alleviate this problem, we used a *hybrid filter-wrapper* algorithm [48]. In this hybrid feature selection model, the features were firstly filtered by *t-*test to find out the statistically significant variables with confidence level of 95%. The 24 variables obtained are then gone through FSS via the wrapper of the classifier of SVM. To alleviate computation cost in wrapping, genetic algorithms was used to find out an informative feature subset. After this step, thirteen features were selected. In the final step, each feature left was examined through the classification test by SVM to remove the features whose contribution to classification accuracy is negligible when it was omitted. Therefore, a compact but highly informative feature subset was obtained. The main advantage of this hybrid approach is that it remains a great part of advantages in wrapper, while reducing the computation cost greatly. We draw a workflow to illustrate the hybrid FSS algorithm in Fig. 3. Table 2 summarized the features removed after each step and the resulted compact features.

**Table 2:** The features removed after Step 2–4 by using the proposed FSS algorithm.

Step 2:	Heterogeneity, Rectangular degree, Elongation, Eccentricity
Step 3:	Fractal dimension, Circularity, Spiculation, Area, Correlation, Inertia, Sum Variance, Sum entropy, Difference Average, Difference Average, Difference Entropy.
Step 4:	Compactness, Solidity, Entropy of Radial Length Distribution, Energy, Sum Average, Information Correlation 2
Final: Output	ADC, Slope, SER, Age, Entropy, Inverse Difference, Information Correlation 1

### Classification Model

The combination of the parameters as a whole could reflect different aspects of lesion properties and is potentially a comprehensive approach to characterize lesion status [49]. The differentiation of malignant from benign lesions was treated as a two-class pattern classification problem. Classical classification algorithms, including support vector machine (SVM) [44], Naïve Bayes (NB), k-nearest neighbors (KNN), and logistic regression (LR) model, were used to evaluate the diagnostic performance of the carefully selected variables [44], [47], [50]. To make an extensive comparison, the derived classier was evaluated through ten-fold cross validation scheme. In the scheme, the data were randomly divided into ten equal subsets. In each experiment, nine subsets were used to construct the predication model and the one left behind was served for testing. The averaged performance after ten times’ experiments was used to evaluate the prognostic capabilities of the selected variables by using measurements including sensitivity, specificity, area under the ROC curve (AUC) and overall accuracy (OA). The hyper-parameters involved in classification models were estimated via five-fold cross validation scheme before testing the corresponding classification algorithm.

## Results

### Diagnostic Performance of each Feature Individually

The proposed feature selection algorithm produced seven features, including one morphology (slope) and three texture (entropy, inverse difference, information correlation 1) parameters, one kinetic parameter (SER), one pathological parameter (age) and ADC. Univariate statistical analyses were conducted to demonstrate the diagnostic capabilities of each feature. All features selected were shown to be statistically different between malignant and benign lesions. Table 3 summarizes the mean and standard deviation and the diagnostic performance on the whole dataset of the seven selected parameters. Among the seven parameters, the diagnostic accuracy of SER was the highest.

**Table 3:** Group mean, *P* values and diagnostic accuracy of selected parameters.

Parameters	Mean±SD		*P* 1 value	Diagnostic^2^ Accuracy	Threshold Value
	Benign	Malignant			
Age	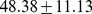	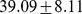	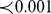	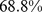	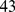
Slope	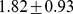	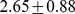	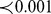	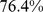	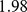
Entropy	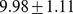	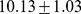	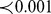	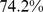	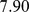
Inverse Difference	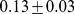	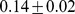	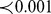	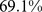	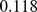
Information Correlation 1	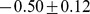	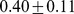	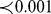	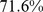	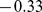
ADC	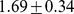	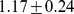	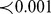	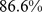	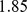
SER^3^	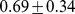	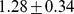	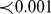	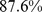	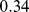

1 Computed with paired-sample t-test.

2 Computed with Receiving Operating Characteristic.

3 Signal enhancement ratio.

### Diagnostic Performance of the Combined Features

In this experiment, we evaluated the diagnostic performance of each individual feature group as well as their combinations through ten-fold cross validation scheme. The whole data were randomly divided into ten equal sized subsets, among which 9 subsets were trained to find out the classifier or optimal cut-off values and the one left behind was used for testing. For the group in which only one feature was selected, univariate analysis was carried out by Receiver Operating Characteristics (ROC). For the feature group which has more than two features, classical classification algorithms including SVM, Naïve Bayes (NB), KNN and Logistic Regression were conducted and their averaged performance was calculated.

The experimental results were summarized in Table.4. When only individual feature group was used, the prediction performance was unsatisfactory. For example, the accuracy is 63.4% by using morphology features. While the accuracy increases to 74.9% by using DWI feature of ADC,. The observation implies two aspects: 1) characterization of the lesion through one-sided methodology was not comprehensive enough. It might have good sensitivity, yet the specificity was poor; 2) ADC is a nice diagnostic factor for discriminating the status of breast mass. Our finding is consistent with earlier results [8], [11], [14]. However, the specificity of ADC was lower than what’s anticipated, making it an unreliable factor in diagnostic practice as a result.

In comparison, when all the well selected features were combined together, the averaged sensitivity, specificity, AUC and accuracy of the classification model dramatically increased to 0.85, 0.89, 0.93 and 90.9%, respectively (Table 4). Among the tested models, although SVM achieved superior performance to other three models in terms of accuracy, the latter ones had comparable results. Therefore, we may draw a conclusion that a full characterization of breast lesion through multi-sided methodologies will produce a high discrimination power.

**Table 4:** Diagnostic performances for differentiating between malignant and benign lesions on various feature sets via 10 fold CV.

Diagnostic Feature	Evaluation Model	Sensitivity	Specificity	Accuracy	AUC
Morphology Features (Slope)	Univariate Threshold	0.74	0.46	63.4%	0.60
Kinetics Features (SER)	Univariate Threshold	0.79	0.52	68.7%	0.66
DWI Feature (ADC)	Univariate Threshold	0.95	0.52	74.9%	0.69
Texture Features (Entropy, Inversedifference Information correlation 1)	SVM	0.51	0.68	72.4%	0.68
	NB	0.56	0.65	72.0%	0.78
	KNN(n = 6)	0.64	0.59	69.8%	0.72
	Logistic Regression	0.57	0.70	74.6%	0.71
	Averaged	0.57	0.66	72.2%	0.72
ADC +Morphology +Kinetics +Pathology	SVM	0.86	0.94	92.4%	0.91
	NB	0.86	0.89	90.5%	0.95
	KNN(n = 6)	0.85	0.871	89.5%	0.94
	Logistic Regression	0.86	0.91	91.3%	0.90
	Averaged	0.85	0.89	90.9%	0.93

## Discussion

The results of our study demonstrate that diagnostic performance can be dramatically improved by incorporating the multi-sided characterizations of the breast lesions on MRI. In particular to the parameter of ADC, it has been shown to be correlated to lesion malignancy due to a high cell density, caused by an increased fraction of signals coming from intracellular water. This parameter, when combined with morphology and enhancement kinetic features, will increase in both specificity and sensitivity in discriminating types of lesions, thus it is promising in providing a supplementary assessment on lesion status.

We carried out a systematical analysis to investigate the potential power in discriminating a fully comprised pictorial characterization of lesions. Our analysis pipeline includes image segmentation, feature extraction, selection and classification model building. The seven features obtained are all shown to be statistically different between the malignant and benign lesions. The combined features were tested extensively through four popular classification models. The finding demonstrates that the combination of the kinetic enhancement data, morphology information and ADC in a systematic model is effective and comprehensive to make an accurate diagnosis on breast masses. We speculate that this could potentially impact clinical management decisions and therapy selection.

## Supporting Information

File S1 **Quantitative measurements of breast lesions.**
(DOCX)
