# Supplementary material for: Diagnosis of Breast Masses from Dynamic Contrast-Enhanced and Diffusion-Weighted MR: A Machine Learning Approach
Source: PLoS One. 2014 Jan 31;9(1):e87387. doi: 10.1371/journal.pone.0087387 (PMC3909149; doi:10.1371/journal.pone.0087387)
Supplement: File S1 — Quantitative measurements of breast lesions. (DOCX) [file pone.0087387.s001.docx]

**File S.1 *Quantitative measurements of breast lesions***

To achieve comprehensive characterization of breast lesion morphology, 24 parameters were computed automatically based on segmented image. The detailed definitions of the parameters are listed below:

*Gray-level co-occurrence matrix (GLCM) and features extracted from GLCM*

Spatial gray-level co-occurrence matrix estimates image properties related to second-order statistics. Each element (*i*,*j*) in GLCM specifies the number of times that the pixel with gray-level value *i* occurred adjacent to a pixel with value *j* at a given offset$(\Delta x,\Delta y)$. Mathematically, the GLCM element over an image S is given as

$p(i,j)=\frac{\#\{[(x,y),(x+\Delta x,y+\Delta y)]\in S|f(x,y)=i,f(x+\Delta x,y+\Delta y)=j\}}{\#S}$，

where represents the number of specific pixel-pairs. If *N* is the number of distinct gray-levels of an image, we denote that

$p_{x}(i)=\sum_{j=1}^{N} p(i,j)$, $i=1,2,\cdots,N$

$p_{y}(j)=\sum_{i=1}^{N} p(i,j)$, $j=1,2,\cdots,N$

$p_{x+y}(k)=\sum_{i=1}^{N} \sum_{j=1}^{N} p(i,j)$, $k=i+j=2,3,\cdots,2N$

$p_{x-y}(k)=\sum_{i=1}^{N} \sum_{j=1}^{N} p(i,j)$, $k=\left|i-j\right|=0,1,\cdots N-1$

The thirteen texture features are then calculated as

*f*_1_: Angular Second Moment

$f_{1}=\sum_{i} \sum_{j} p(i,j)^{2}$.

*f*_2_: Contrast

$f_{1}=\sum_{k=0}^{N-1} k^{2}p_{x-y}(k)$.

*f*_3_: Correlation

$f_{4}=\frac{1}{\sigma_{x}\sigma_{y}}\sum_{i=1}^{N} \sum_{j=1}^{N} (ij)[p(i,j)-\mu_{x}\mu_{y}]$,

where$\mu_{x}$and$\sigma_{x}$are the mean and standard deviations of$p_{x}$, respectively; $\mu_{y}$and$\sigma_{y}$ are the mean and standard deviations of$p_{x}$, respectively.

*f*_4_: Inverse Difference Moment

$f_{4}=\sum_{i} \sum_{j} \frac{1}{1+(i-j)^{2}}p(i,j)$.

*f*_5_: Sum Average

$f_{5}=\sum_{k=2}^{2N} kp_{x+y}(k)$.

*f*_6_: Sum Variance

$f_{6}=\sum_{k=2}^{2N} (k-f_{5})^{2}p_{x+y}(k)$.

*f*_7_: Sum Entropy

$f_{7}=-\sum_{k=2}^{2N} p_{x+y}(k)\log(p_{x+y}(k))$.

*f*_8_: Entropy

$f_{8}=-\sum_{i} \sum_{j} p(i,j)\log(p(i,j))$.

*f*_9_: Difference Average

$f_{9}=\sum_{i=1}^{N} (i-\mu)^{2}p_{x}(i)$,

where$\mu$is the average of $p(i,j)$.

*f*_10_: Difference Variance

$f_{10}=\sum_{k=0}^{N-1} (k-d)p_{x-y}(k)$,

where $d$is the mean of $p_{x-y}$*.*

*f*_11_: Difference Entropy

$f_{11}=-\sum_{k=0}^{N-1} p_{x-y}(k)\log(p_{x-y}(k))$.

*f*_12_: Information Measure of Correlation 1

$f_{12}=\frac{f_{9}+\sum_{i} \sum_{j} p(i,j)\log(p_{x}(i)p_{y}(j))}{\max(-\sum_{j} p_{x}(i)\log(p_{x}(i)),-\sum_{j} p_{y}(i)\log(p_{y}(i)))}$.

*f*_13_: Information Measure of Correlation 2

$f_{13}=\sqrt{1-\exp(-2(E-f_{8}))}$,

where $E=-\sum_{i} \sum_{j} p_{x}(i)p_{y}(j)\log(p_{x}(i)p_{y}(j))$.

*p*_1_: Compactness

$p_{1}=\frac{P^{2}}{4\pi S}$,

where $P$and$S$ are the perimeter length and area for a given breast MRI lesion contour, respectively.

*p*_2_: Spiculation

$p_{2}=\frac{1}{N}\sum_{i=1}^{N} \left|r_{i}-r_{i+1}\right|$,

where $N$ is the number of pixels on the lesion contour and $r_{i}$ is the individual radial length.

*p*_3_: Extent

$$p_{3}=\frac{S}{S_{\text{box}}}$$

where $S_{box}$is the area of the smallest rectangle containing the given lesion contour.

*p*_4_: Elongation

$$p_{4}=\frac{\min(H,L)}{\max(H,L)}$$

where$H$and$L$are the vertical and horizontal lengths of the smallest rectangle containing the given lesion contour.

*p*_5_: Solidity

where $S_{convex}$ is the area of the smallest convex polygon that can contain the given lesion contour.

*p*_6_: Circularity

$$p_{6}=\frac{1}{N-1}\sum_{i=1}^{N} r_{i}-\mu_{r}$$

where $\mu_{r}$is the average of$r_{i}$.

*p*_7_: Entropy of radial length distribution

$$p_{7}=-\sum p(r_{i})\log(p(r_{i}))$$

where $p(r_{i})$is the probability density of a given$r_{i}$.

**Heterogeneity**: Fraction of pixels that deviate more than a certain range (10% default) from the average intensity.

**Fractal dimension**: Minkowski dimension of the boundary of the node, computed by box-counting method.

**Minkowski dimension**: a way of determining the [fractal dimension](http://en.wikipedia.org/wiki/Fractal_dimension) of set S in a [Euclidean space](http://en.wikipedia.org/wiki/Euclidean_space) $R^{n}$. It is estimated by limit of $\lim_{\varepsilon\to0} \frac{N_{\varepsilon}}{1/\varepsilon}$, where $N(\varepsilon)$is the number of boxes of side length $\varepsilon$required to cover the set S.

**Area:** Pixel numbers of the lesion region.

**Eccentricity:** Scalar that specifies the eccentricity of the ellipse that has the same second-moments as the region. The eccentricity is the ratio of the distance between the foci of the ellipse and its major axis length.
